# Amniocentesis, Maternal Psychopathology and Prenatal Representations of Attachment: A Prospective Comparative Study

**DOI:** 10.1371/journal.pone.0041777

**Published:** 2012-07-25

**Authors:** Wissam El-Hage, Julie Léger, Aude Delcuze, Bruno Giraudeau, Franck Perrotin

**Affiliations:** 1 Clinique Psychiatrique Universitaire, CHRU de Tours, France; 2 INSERM U930 ERL, Université François Rabelais de Tours, France; 3 INSERM CIC 0202, CHRU de Tours, France; 4 Fetal Medicine Unit, CHRU de Tours, France; Catholic University of Sacred Heart of Rome, Italy

## Abstract

**Background:**

The aim of the study was to characterize the maternal dimensions of anxiety, depression and prenatal attachment in women undergoing an amniocentesis.

**Methodology/Principal Findings:**

A prospective observational study was conducted. Women were referred to early amniocentesis for increased nuchal translucency, elevated biochemical markers or advanced maternal age. All participants had 3 prenatal (16–18, 20–24, 30–34 weeks of gestation) and one postnatal (30–45 days) interviews reviewing for demographic, medical, and psychiatric information (STAI State-Trait Anxiety Inventory; EPDS: Edinburgh Postnatal Depression Scale; IRMAG: Interview of Maternal Representations of Attachment during pregnancy). We investigated 232 pregnant women who undergone an amniocentesis compared with 160 pregnant controls. Following the procedure, the amniocentesis group experienced transiently significantly higher levels of state-anxiety on the STAI (44.6 *vs.* 39.3) and depression as measured by the EPDS (9.4 *vs.* 6.3) than the controls. Overall in both groups, the maternal representations of attachment were well integrated and balanced, but the amniocentesis group experienced significantly more mother-directed representations.

**Conclusions/Significance:**

Amniocentesis is associated with higher affective adaptive reactions that tend to normalize during the pregnancy, with overall preserved maternal fetal representations of attachment.

## Introduction

Maternal fetal attachment is the extent to which women engage in behaviors that represent an affiliation and interaction with her unborn child [1], [2]. Prenatal attachment increases throughout gestation [3]–[8] and fetal development is usually accompanied with changes in maternal representations. Raphael-Leff [9] described three different maternal psychological styles: *i)* the facilitating mother who tries to adapt to the child’s biological rhythms and behavior, *ii)* the regulating mother who tries to have the child to adapt to her own routines, and *iii)* the reciprocator mother who lives the pregnancy in a vague and disrupted way. After 7 months of pregnancy, maternal representations begin to decline in intensity, which in one theoretical frame is regarded as an undoing of their positive representations to prevent disappointment [10] and, in a more neurobiological frame, may reflect consolidation of the neural circuitry of attachment systems [11].

Subjective experience of childbirth, history of obstetric complications, social isolation and dependent personality disorder and high level of stress-anxiety after childbirth are significant predictive factors of postpartum psychopathology [12]. Several studies suggested the predictive value of maternal representations and the continuity between maternal prenatal and postnatal attachment [13]–[17]. In a recent meta-analysis, Yarcheski et al. [18] identified only three predictors of maternal fetal attachment, with moderate effect sizes: social support, prenatal diagnosis and gestational age. Moreover, ultrasound imaging seems to reduce the level of anxiety and to hasten bonding [19], [20]. Maternal anxiety indicators have also been linked to higher levels of fetal motor activity [21], which may affect maternal representations of the fetus. However, to our knowledge, no study explored how anxiety level may directly affect maternal fetal attachment.

Amniocentesis for detection of chromosomal anomalies is usually offered at the beginning of the second trimester to woman with advances maternal age (≥38 years), a personal history of chromosomal anomaly or in case of positive chromosomal abnormality screening (increased nuchal translucency or biochemical markers). According to the studies, amniocentesis was associated to increased [22]–[24] or decreased [25] levels of anxiety. These discrepancies are due to methodological limitations such as small sample size, inconsistent measurements of maternal-fetal attachment during gestational periods, and cross-sectional designs [18], [26], [27]. Nevertheless, several studies supported that the amniocentesis procedure impact negatively prenatal attachment [28], delaying bonding until definitive favorable results are received [28], [29].

Thus, we have chosen a prospective design for this study comparing pregnant women who undergone an amniocentesis to pregnant controls. The main objective of this study was to investigate the relationship between amniocentesis for fetal karyotype and psychopathological dimensions such as maternal prenatal representations of attachment, anxiety and depression levels.

## Results

### Subjects

Table 1 summarizes the social, professional and psychopathological characteristics of the participants. Compared to the control group, women from the amniocentesis groups were significantly older at the time of the investigation, older of 4 years at their first childbirth, more active, more highly educated and fewer were nulliparous. Significantly more women from the amniocentesis groups suffered from history of child abuse, anxiety or depression in the adolescence, and alcohol consumption before and during the current pregnancy. No significant difference was found between the groups concerning medication use, other addictions, trait anxiety levels and other traumatic events.

**Table 1:** Maternal characteristics of the amniocentesis group “Amnio” (n = 222) and controls (n = 120).

	Amnio* (n = 222)	Control*(n = 120)	p-value
Age at baseline	34.9±5.1	28.2±5.0	<0.001
Indication of amniocentesis			
High maternal age	79 (36.6)	–	–
High serum marker	148 (66.7)	–	–
Thick nape	5 (2.3)	–	–
Cohabitation	206 (93.2)	104 (96.3)	0.260
Geographical origin			
France	205 (92.8)	94 (87.0)	0.088^F^
Europe	5 (2.2)	4 (3.7)	
Africa	7 (3.2)	10 (9.3)	
Asia	2 (0.9)	0	
America	2 (0.9)	0	
Professionally active	188 (85.1)	68 (63.0)	<0.001
Number of years of studies	14.9±2.7	14.1±2.6	0.009
Tiring working conditions	78 (35.3)	32 (29.6)	0.306
Precarious housing	5 (2.3)	0	0.175^F^
Parent's death during childhood	11 (5.1)	8 (7.3)	0.427
Child abuse during childhood	18 (8.4)	2 (1.9)	0.021
Eating disorders during adolescence	17 (7.9)	7 (6.5)	0.637
Depression or anxiety during adolescence	19 (8.9)	3 (2.8)	0.041
Conjugal violence	9 (4.2)	1 (0.9)	0.174^F^
Primiparous	73 (33.0)	48 (44.4)	0.044
Age at first child's birth	28.5±5.4	24.8±4.2	<0.001
Gravidity			
0	8 (3.6)	4 (3.7)	0.012^T^
1	54 (24.4)	36 (33.3)	
2	62 (28.1)	35 (32.4)	
3	52 (22.6)	21 (19.5)	
4 and over	47 (21.3)	12 (11.1)	
History of abortion or termination	67 (30.3)	30 (27.8)	0.635
History of amniocentesis	29 (13.1)	3 (2.8)	0.003
Infertility	13 (5.9)	3 (2.8)	0.219
Tobacco use before pregnancy	70 (31.8)	42 (38.9)	0.204
Tobacco use during pregnancy	32 (14.6)	22 (20.4)	0.181
Alcohol use before pregnancy	90 (40.9)	15 (13.9)	<0.001
Alcohol use during pregnancy	20 (9.1)	1 (0.9)	0.005
Use of substitution products	1 (0.5)	1 (0.9)	0.551^F^
Medications use during pregnancy	43 (19.7)	14 (13.0)	0.130
Type of medications			
Psychotropic drugs	6 (14.0)	0	0.320^F^
Others	39 (86.0)	14 (100)	

* Data are reported as mean ± standard deviation or n (%).

F Fisher’s exact test.

T Cochran-Armitage test for trend.

Table 2 summarizes the social, professional, and psychopathological characteristics of the partners of the participants. Compared to the partners from the control group, the partners from the amniocentesis groups were significantly more active, with a higher level of education, more addictive to alcohol and less addictive to tobacco.

**Table 2:** Characteristics of the partners of the participants from the amniocentesis group “Amnio” (n = 222) and the controls (n = 120).

	Amnio* (n = 222)	Control*(n = 120)	p-value
Geographical origin			
France	191 (88.4)	95 (88.8)	0.866^F^
Europe	9 (4.1)	3 (2.8)	
Africa	14 (6.5)	9 (8.4)	
Asia	1 (0.5)	0	
America	1 (0.5)	0	
Professional activity	210 (97.2)	98 (91.6)	0.045^F^
Number of years of studies	14.4±2.9	13.5±3.2	0.015
Tobacco consumption before pregnancy	69 (32.1)	52 (48.6)	0.004
Alcohol consumption before pregnancy	120 (55.8)	39 (36.5)	0.001
Drug consumption before pregnancy	3 (1.4)	3 (2.8)	0.403

* Data are reported as mean ± standard deviation or n (%). ^F^Fisher's exact test.

For analysis, the “amniocentesis” group was subdivided into two subgroups, the “routine amniocentesis” group including women who were routinely offered amniocentesis for advanced maternal age (n = 79) and the “indicated amniocentesis” group (n = 153) with patients who were counseled regarding amniocentesis for elevated biochemical markers (n = 148) or increased nuchal translucency (n = 5). The “control” group included 160 pregnancies with no indication of amniocentesis.

### Obstetrical Characteristics

The ongoing pregnancy was normal for the majority of the cases. However 6 patients had premature delivery, 46 pregnancies ended by caesarean section, 33 had operative delivery and postpartum hemorrhage occurred for 12 patients. No significant difference was found between the groups for the obstetrical complications. However, significantly more women choose to breastfeed in the amniocentesis group (69% *vs.* 51%, p = 0.004).

### Maternal Anxiety Symptoms

**Figure 1:**
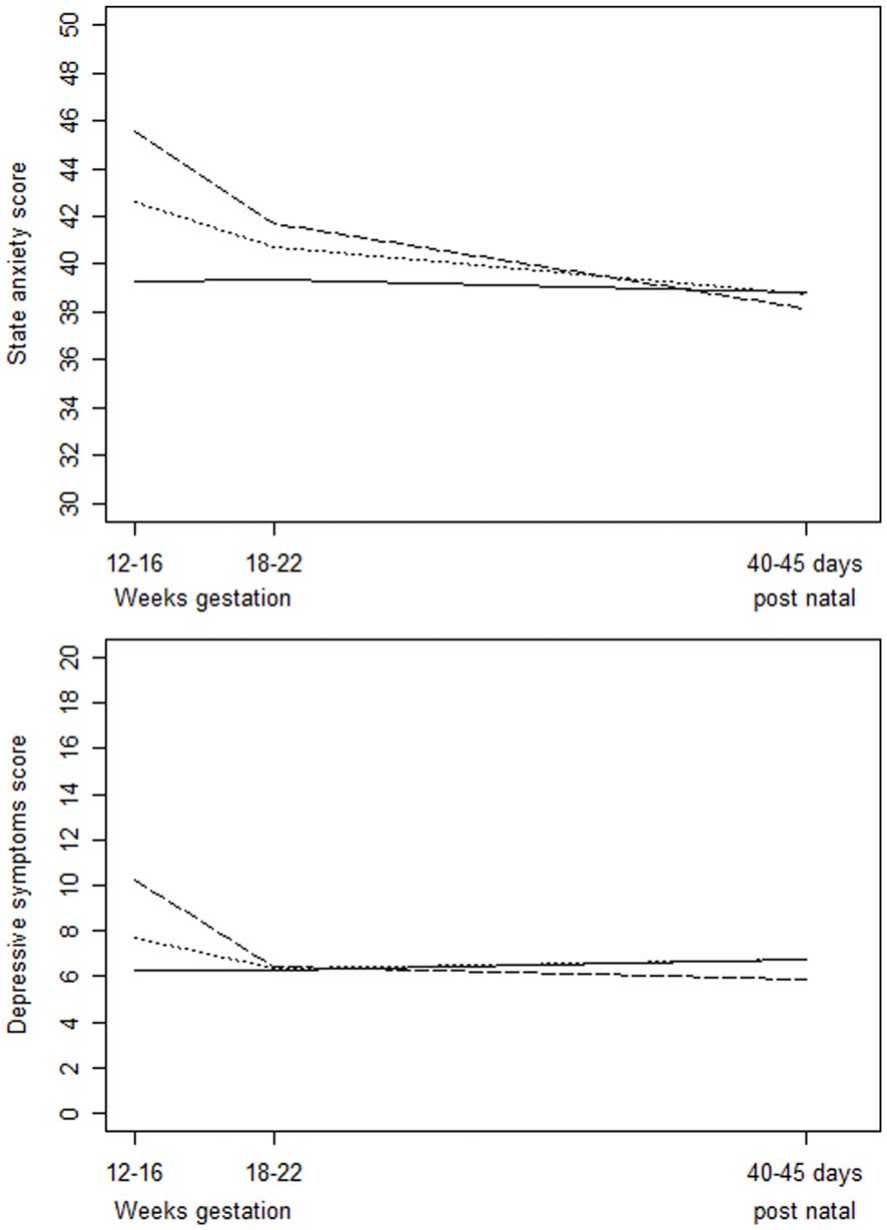
Maternal state anxiety and depressive symptoms course during the pregnancy up to 40–45 days post natal. Solid line is for control group, dotted line is for “routine amniocentesis” group and long-dashed line is for “Indicated amniocentesis” group.

Women from the “indicated amniocentesis” group experienced significantly higher levels of state-anxiety (p = 0.002) (Figure 1), mostly immediately after the amniocentesis procedure (45.6±11.0) than in the control group (39.3±11.4). However difference in anxiety levels tended to decrease over the course of the pregnancy, and was slighter at 20–24 weeks of gestation (41.7±11.3 *vs.* 39.4±12.1, p = 0.86) and was no more significant in the postpartum period (p = 0.57). In controls, the anxiety levels remained dramatically unchanged over the course of the pregnancy. Further analysis showed significant main effect of visit (p<0.001) but no significant effect of group and significant visit by group interaction effect (p<0.001). The interaction was accounted for by patients from the amniocentesis group showing higher scores of anxiety than healthy controls, at visits 1 and 2 (Table 3).

**Table 3:** Between-group differences in state-anxiety scores (STAI).

Effect	Estimate	SE*	p-value
Intercept	38.24	1.41	<0.001
V2 *vs.* V1	0.22	1.26	0.860
V4 *vs.* V1	−0.84	1.48	0.571
Propensity score	2.65	2.39	0.268
Routine amniocentesis*vs.* controls	1.83	2.23	0.413
Indicated amniocentesis*vs.* controls	5.43	1.71	0.002
V2 * Routine amniocentesis*vs.* controls	−1.38	2.34	0.556
V2 * Indicated amniocentesis*vs.* controls	−4.57	1.73	0.009
V4 * Routine amniocentesis*vs.* controls	−3.04	2.37	0.200
V4 * Indicated amniocentesis*vs.* controls	−6.87	1.93	<0.001

* SE: Standard error; V1: Visit 1; V2: Visit 2; V4: Visit 4.

### Maternal Depression Symptoms

In the control group, the depression mean scores remained unchanged over the course of the pregnancy (Figure 1). In the “indicated amniocentesis” group, depression mean scores were significantly higher (10.2±5.1) than in the “routine amniocentesis” group (7.7±6.2) and the “control” group (6.3±4.3), immediately after the amniocentesis procedure. Hereafter, depression mean scores decreased in both “amniocentesis” groups. After 20–24 weeks of gestation, there was no difference between the three groups.

Further analysis showed significant effect of visit (p<0.001) and significant visit by group interactions (p<0.001) but no significant effect of group (p = 0.26). The interaction was accounted for by patients from the amniocentesis group showing higher scores of depression than healthy controls, at 16–18 weeks of gestation (Table 4).

**Table 4:** Between-group differences in depression scores (EPDS).

Effect	Estimate	SE*	p-value
Intercept	6.34	0.66	<0.001
V2 *vs.* V1	−0.003	0.58	0.996
V4 *vs.* V1	0.40	0.71	0.573
Propensity score	−0.09	1.11	0.937
Routine amniocentesis*vs.* controls	1.40	1.04	0.180
Indicated amniocentesis*vs.* controls	3.96	0.80	<0.001
V2 * Routine amniocentesis*vs.* controls	−0.77	1.06	0.470
V2 * Indicated amniocentesis*vs.* controls	−3.88	0.79	<0.001
V4 * Routine amniocentesis*vs.* controls	−1.20	1.14	0.292
V4 * Indicated amniocentesis*vs.* controls	−4.63	0.93	<0.001

* SE: Standard error; V1: Visit 1; V2: Visit 2; V4: Visit 4.

### Maternal Post-traumatic Symptoms

In the “amniocentesis” group, the prevalence of acute stress disorder was 8.5% after amniocentesis and 6.9% after childbirth. Women from the “amniocentesis” (n = 124) and the “control” (n = 64) groups completed the PPQ in order to assess the prevalence of post-traumatic stress symptoms during the first 45 days after giving birth. No significant difference was found between the “amniocentesis” (2.1±2.4) and the “control” (2.3±2.4) groups in the total PPQ scores at 45 days after giving birth (p = 0.51).

In all groups, the DES mean scores remained stable over the course of the pregnancy. There was no significant difference between the “amniocentesis” and the “control” groups at V1 (11.3±8.9 *vs.* 12.2±11.6), at V2 (10.5±9.7 *vs.* 11.3±12.1) and at V4 (12.2±10.3 *vs.* 10.9±11.3). The analysis did not show significant effect of visit (p = 0.34), of group (p = 0.54) or visit by group interactions (p = 0.87).

### Antenatal Maternal Representations of Attachment

The distribution of the various types of maternal representations was significantly (p<0.001) different between the three groups (Table 5). Compared to controls, women from the “amniocentesis” group had overall normally integrated and balanced representations of attachment to their unborn child, but significantly more frequently directed on oneself than on the child. Moreover, two patients from the “indicated amniocentesis” group experienced reduced and disengaged maternal representations with fear.

**Table 5:** Antenatal maternal representations of attachment by group.

Maternal representations	Amniocentesis		Controls	Total
	Routine	Indicated		
Integrated-balanceddirected on child	30 (63.8)	80 (69.6)	58 (84.1)	168
Integrated-balanced directedon oneself	17 (36.2)	33 (28.7)	11 (15.9)	61
Limited-disengaged with fear	0 (0)	2 (1.7)	0 (0)	2
Total	47	115	69	231

* Data are reported as n (%) or n.

Women from all “control”, “routine amniocentesis” and “indicated amniocentesis” groups had similar representations of themselves-as-women concerning richness of perceptions (p = 0.49), intensity of involvement (p = 0.44), coherence (p = 0.17), social dependence (p = 0.55) and immersion in fantasy (p = 0.72), but had significantly different representations with a reduction of intensity for openness to change (p = 0.02) and for differentiation (p = 0.04). Similarly, concerning the maternal representations of the child, they did not differ from the others on their richness of perceptions (p = 0.29), intensity of involvement (p = 0.46), coherence (p = 0.29), differentiation (p = 0.46), social dependence (p = 0.11) and immersion in fantasy (p = 0.75), but differed only in their openness to change (p = 0.01), which was less intense in the “indicated amniocentesis” group.

## Discussion

In this study, the maternal representations were overall normally integrated and balanced in all groups. Nevertheless, women that experienced an amniocentesis procedure had clearly more representations directed on oneself than on the fetus. Amniocentesis, which was indicated for increased nuchal translucency or elevated biochemical markers, was associated with significant increase of anxiety and depression symptoms. This difference was transient, tended to decrease over the course of the pregnancy and reached no significant level at the postpartum period. Interestingly, we found that women initiating breastfeeding were significantly more prevalent in the amniocentesis group than in the control group. The difference in initiating breastfeeding could be understood as a maternal positive adaptive response to the negative stress induced by the amniocentesis procedure. However, this hypothesis cannot be confirmed here as breastfeeding is usually more frequent in women with higher level of education [30].

This is the first large study showing that overall the maternal representations of attachment were not negatively impacted by the amniocentesis procedure. Our results differ from previous studies but confirm Tercyak’s study [24]. Undergoing amniocentesis did not considerably alter maternal representations of attachment, even if more representations were directed on oneself than on the fetus. These differences could be understood as an adaptive mechanism to the fear of fetal loss in these women, probably in relation with their own personality or personal history. No woman from our sample had restricted-disengaged or not integrated/ambivalent representations. This unexpected result is probably due to the possibility offered to women to undergo termination of pregnancy. In fact, French law allows termination of pregnancy with no gestational age limit when severe fetal abnormalities are observed. Here, from the initially recruited sample, four women decided termination of pregnancy after Down syndrome was diagnosed. We presume that if these women did not have the possibility to interrupt the pregnancy, their maternal-fetal representations of attachment would have been probably more disengaged or ambivalent compared to those who had normal karyotype results. Moreover, the clinical interviews showed that most of the pregnancies have been desired and planned, and nearly all mothers believed that they will be good mothers for their own infant.

Women’s immediate anxious and depressive reactions after the amniocentesis could be related to the risk that something could be genetically wrong with their fetus, which could result in a decision to terminate the pregnancy. During the waiting period, between amniocentesis procedure and its results, women act as if they were withdrawing from their pregnancies while the diagnosis is pending. They strive to maintain an emotional distance and to avoid attaching to the pregnancy until the good health of their fetus is confirmed. Moreover, moderately increased anxiety scores may reflect increased arousal that is, according to the decision making theory, necessary for actively engaging in making a choice between options with serious consequences [31]. This explanation is supported by the fact that this affective reaction vanishes after receiving reassuring fetal results, within two or three weeks.

Interestingly, women who underwent amniocentesis for advanced maternal age did not have significant differences with controls. They were probably less anxious than the complex amniocentesis group because they were not screened positive for increased risk for Down syndrome (increased nuchal translucency, elevated biochemical markers).

### Limitation

One important limitation of the present study is the relatively small number of women enrolled in the control group. This limitation can be explained by encountered difficulties in recruiting normal pregnancies, especially due to the fact that the fetal medicine unit where the recruitment took place is referral center dedicated mostly to manage fetal defects and high-risk pregnancies. Another major limitation is the high level of missing data, particularly in the control group. This is a common problem in modern health research and particularly in all cohort studies having repeated measures over time, which are subject to attrition. Women drop out because they had no medical benefit from their participation or for other mostly unknown reasons. So there was a self-selection bias but we took into account these missing data for the statistical analysis. Despite these limitations, this study remains an original prospective well controlled study with larger sample size than previous ones.

Previous studies showed high correlation between prenatal and postnatal attachment. Our study screened only for prenatal maternal representations of attachment. Future studies should address the link between prenatal maternal representations of attachment and long-term postnatal impact on maternal attachment and the child’s development.

### Recommendation

The practitioners are used to inform the patients that the procedure of amniocentesis can be associated with a risk of miscarriage. We recommend them also to consider the psychological aspects related to this procedure and more particularly to the waiting period, between amniocentesis procedure and its results. Thus, the practitioners should discuss the possible risk for the pregnant women to develop temporary symptoms of anxiety or depression after the procedure of amniocentesis. The information should be delivered before the procedure and attention must be paid to the wording with which the practitioner will inform the patients that the amniocentesis may be associated with emotional adaptive reactions, reassuring them that these symptoms usually tend to normalize during the pregnancy. In case of persistent affective symptoms, a support from mental health professionals should be considered.

## Methods

### Study Design

This is a cohort study. Both women exposed and not exposed to amniocentesis were included between 16 and 18^+6^ weeks of gestation and followed until 30–45 days postpartum.

The study was monocentric. All women were included in the fetal medicine unit of a tertiary referral center offering all prenatal diagnosis facilities (CHRU de Tours, France) with approximately 500 amniocentesis performed each year. Exposed patients were recruited just after their amniocentesis and controls were recruited after their first-trimester scan. Each participant had three prenatal visits (V1: 16–18^+6^ weeks of gestation; V2: 20–24^+6^ weeks of gestation; V3: 30–34^+6^ weeks of gestation) and one postpartum visit (V4; 30–45 days postpartum).

**Figure 2:**
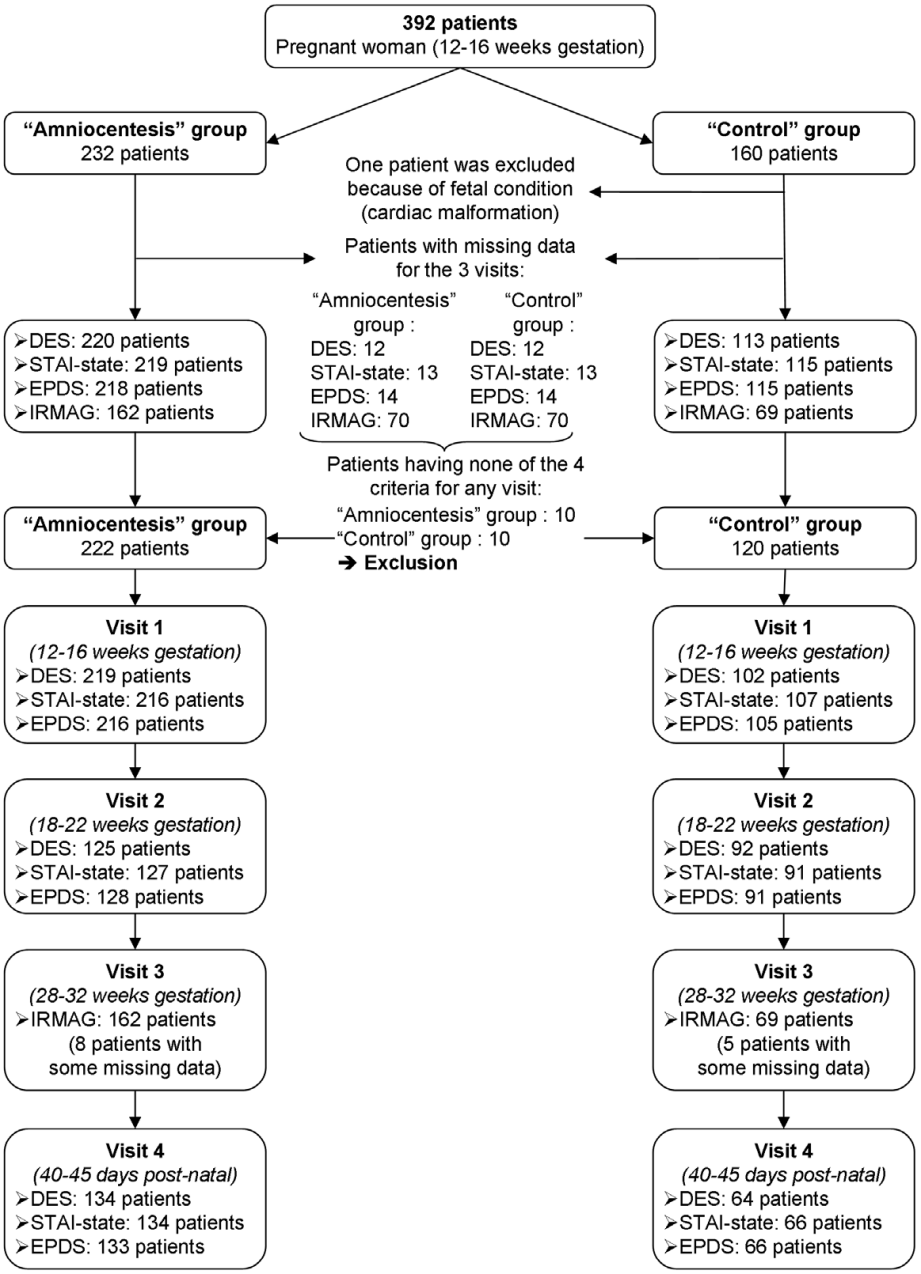
Clinical trial profile of the study.

The current study analyzed data of 392 pregnant women (Figure 2). One patient was excluded due to the discovery during second trimester of a severe fetal defect (cardiac malformation). Participants with missing data for the three visits were also excluded from further analysis.

### Participants

This study included only singleton pregnancies with no maternal or fetal condition. We excluded participants with preterm delivery, legal incapacity to consent or to understand the study, history of fetal malformation, fetal death or medical termination of pregnancy. Were secondarily excluded participants with detection of fetal malformation after the first trimester, or late discovery of maternal pathology requiring monitoring.

For women who had an amniocentesis, participation to the study was systematically proposed whatever the indication of amniocentesis (advanced maternal age, elevated biochemical markers or increased nuchal translucency). The study was also systematically proposed to control pregnant women recruited from the same unit.

### Ethical Issues

Participants were given full details about the experimental protocol and gave their informed consent before the beginning of the experiments. This study conforms to the Code of Ethics of the World Medical Association (Declaration of Helsinki) and was approved by the local Ethical Committee.

### Variables

Data extracted from the medical records comprised maternal age, obstetrical and medical history, details about the management and the course of the current pregnancy, the delivery and the baby’s health. Clinical interviews provided information concerning the socio-demographic status and addiction habits before and during pregnancy. Maternal addictions (cigarettes, alcohol and cannabis) were assessed dichotomously (yes or no), regardless of the quantitative aspects. During V1, V2 and V4, mothers completed self-reported questionnaires evaluating life stress events, anxiety, depression, and dissociation symptoms. During V3, we assessed maternal representations of attachment (Table 6).

**Table 6:** Study plan.

	Visit 1	Visit 2	Visit 3	Visit 4
	16–18 weeks of gestation	20–24 weeks of gestation	30–34 weeks of gestation	30–45 dayspost-natal
Socio-demographics	X			
Addiction habits	X	X		X
Life stress events, CAPS	X			
Anxiety symptoms, STAI	X	X		X
Depression symptoms, EPDS	X	X		X
Dissociation symptoms, DES	X	X		X
Maternal representations, IRMAG			X	
Obstetrical data				X
Post-traumatic symptoms, PPQ				X

CAPS: Clinical Administered interview for the diagnosis of Post-traumatic stress disorder; DES: Dissociative Experiences Scale; EPDS: Edinburgh Postnatal Depression Scale; IRMAG: Interview of Maternal Representations of Attachment during pregnancy; PPQ: Perinatal PTSD Questionnaire; STAI: State-Trait Anxiety Inventory.

We assessed the history of traumatic events, using an exhaustive list of life events extracted from the CAPS (interview for the diagnosis of PTSD, post-traumatic stress disorder) [32], the postnatal post-traumatic acute symptoms related to childbirth (PPQ, Perinatal PTSD Questionnaire) [33] and the dissociation symptoms (DES, Dissociative Experiences Scale) [34]. Current and usual anxiety levels were assessed using the French version of the State-Trait Anxiety Inventory (STAI) [35]. Maternal depressive symptoms were evaluated with the French version of the Edinburgh Postnatal Depression Scale (EPDS) [36], [37].

Maternal representations of attachment were assessed with the Interview of Maternal Representations of Attachment during pregnancy (IRMAG) [7]. The IRMAG is a semi-structured interview administered, by a trained psychologist, between 30 and 34 weeks of gestation. It includes 41 questions and lasted 60–90 minutes. It was entirely registered and typewritten before a double quotation by two different trained psychologists. The IRMAG explores in a pregnant woman her desire of motherhood, her personal feelings to the announcement of the pregnancy and related changes in her personal life, her couple and with regard to her own mother, space to the internal child, future prospects about her own characteristics as a mother, the imagined characteristics of the child, and the historical perspective and characteristics of her own mother. Moreover, the narrative structure of the IRMAG was analyzed extracting 7 dimensions: richness of perceptions, openness to change, intensity of involvement, coherence, differentiation, social dependence, and immersion in fantasy. Consequently and in each participant, these dimensions allow characterizing the maternal representations of attachment: integrated-balanced (limited, directed on herself, or directed on child), limited-disengaged (stressed, directed on herself, or with fear) or not integrated-ambivalent (vague, inversion of role, or absorbed by herself).

### Statistical Analysis

In the present study, we selected three outcomes to be compared between groups: the mean scores of the DES, the STAI-state and the EPDS. Sample size calculation would have supposed to formulate three hypotheses (one for each outcome), and moreover to have baseline data for each of these outcomes. This seemed cumbersome and we actually planned to recruit at least 100 women per group, which is indeed an arbitrary sample size. We recognize this can be viewed as a debatable choice, but, as recently acknowledged, "Conventional power calculations provide precise sample sizes - but only by using precise assumptions. Accurately specifying such precise assumptions is challenging for any study and almost always impossible for early clinical studies" [38]. Because of very few prior data when the present study was planned, we therefore fixed an arbitrary sample size.

Individual characteristics were described with means ± standard deviations (SD) for quantitative variables or numbers and percentages for qualitative variables. Groups were compared thanks to Student t-test, chi-square test (or Fisher’s exact test if conditions were not respected) and Cochran-Armitage test for trend for ordinal data.

Prior to any analysis, a propensity score [39] was estimated for each patient. Formally, the propensity score is the probability of assignment to a particular treatment given a vector of observed covariates. In our study, this score is the probability for a patient to have an amniocentesis conditionally to her baseline characteristics. In observational studies, treated and control groups may have large differences on their covariates, and these differences can lead to biased estimates of treatment effects. Then propensity score can be used to balance the covariates in the two groups, and so reduce this bias. The propensity score was estimated using logistic regression and we introduced it as an adjustment variable in our statistical models. Logistic regression excluded patients with missing data so they were treated as follows. For continuous variables: missing data were replaced by group’s mean. For binary data, for each missing data a number between 0 and 1 was randomly generated from a Bernoulli distribution with parameter equal to the observed probability. For qualitative data, for each missing data a number between 0 and 1 was randomly generated from hypergeometric distribution where parameters were estimated on available data. This imputation’s procedure was repeated 1,000 times and for each imputation a propensity score was estimated. The “final” propensity score is the mean score of the 1,000 propensity scores.

The DES, STAI state and EPDS scores were compared between groups thanks to linear mixed models to take intra-subject correlation into account. IRMAG was composed of various parameters. For the type of maternal representations, amniocentesis and control groups were compared using Fisher’s exact test. Perceived individual characteristics of the unborn infant, the subject herself and the infant’s father, maternal characteristics of self-as-woman and of own mother were compared by groups with Student t-test.

Data about obstetrical complications and delivery were compared between groups with chi-square test or Fisher’s exact test. All analyses were performed using SAS Version 9.1 software for Windows (SAS Institute, Inc., Cary, NC, USA) and charts were done using R version 2.7.2 [40].

### Conclusion

Amniocentesis is associated with higher affective adaptive reactions that tend to normalize during the pregnancy, with overall preserved maternal fetal representations of attachment. Further investigations should also consider how amniocentesis impacts not only the pregnant women but also their partners and their relationships. Furthermore, the progress of research in human genetics [41], [42], which holds great promise for the health, requires also studying the impact of such invasive procedures on the stress hormones (gene x environment interactions) on the epigenome and how this interaction impacts the child’s neurodevelopment.
